# Bromide of Calcium

**Published:** 1872-01

**Authors:** 


Bromide of Calcium.—In a note to the New York Medical Journal, December, 1871, Dr. Wm. A. Hammond calls attention to some advantages of the bromide of calcium in certain conditions of the nervous system. He contends that the effect can be obtained more promptly than when either of the other bromides are used, owing to its instability, the bromide being more readily set free than in the other combinations; also, that it is more decidedly and promptly hypnotic than the other preparations, and consequently more beneficial in cases of delirium tremens, or insomnia from other causes. He gives it in from fifteen to thirty grains or more for an adult. He says:—"I gave a gentleman, who, owing to business anxieties had not slept for several nights, and who was in a state of great excitement, a dose of thirty grains. He soon fell into a sound sleep, which lasted for seven hours. The next night, as he was wakeful, I gave him a like dose of bromide of potassium; but it was without effect, and he remained awake the whole night. The subsequent night he was as indisposed to sleep as he ever had been, but a dose of thirty grains of bromide of calcium gave him eight hours sound sleep, and he awoke refreshed, and with all unpleasant cerebral symptoms—pain, vertigo, and confusion of ideas—entirely gone. In a number of other instances, a single dose has sufficed to induce sleep—a result which very rarely follows the administration of one dose of any of the other bromides. In those exhausted conditions of the nervous system attended with great irritability—such as are frequently met with in hysterical women, and which are indicated by headache, vertigo, insomnia, and a mental condition of extreme excitement—bromide of calcium has proved, in my hands, of decided service. Combined with the syrup of the lacto-phosphate of lime, it scarcely leaves anything to be desired. An eligible formula is: ℞.—Calcii bromidi ℥ i, syrup lact. phos. calc. ℥ iv. M. ft. sol. Dose, a tea-spoonful three times a day in a little water."

